# Dairy *Streptococcus thermophilus* improves cell viability of *Lactobacillus brevis* NPS-QW-145 and its γ-aminobutyric acid biosynthesis ability in milk

**DOI:** 10.1038/srep12885

**Published:** 2015-08-06

**Authors:** Qinglong Wu, Yee-Song Law, Nagendra P. Shah

**Affiliations:** 1 Food and Nutritional Science, School of Biological Sciences, The University of Hong Kong, Pokfulam Road, Hong Kong

## Abstract

Most high γ-aminobutyric acid (GABA) producers are *Lactobacillus brevis* of plant origin, which may be not able to ferment milk well due to its poor proteolytic nature as evidenced by the absence of genes encoding extracellular proteinases in its genome. In the present study, two glutamic acid decarboxylase (GAD) genes, *gadA* and *gadB*, were found in high GABA-producing *L. brevis* NPS-QW-145. Co-culturing of this organism with conventional dairy starters was carried out to manufacture GABA-rich fermented milk. It was observed that all the selected strains of *Streptococcus thermophilus*, but not *Lactobacillus delbrueckii* subsp. *bulgaricus*, improved the viability of *L. brevis* NPS-QW-145 in milk. Only certain strains of *S. thermophilus* improved the *gadA* mRNA level in *L. brevis* NPS-QW-145, thus enhanced GABA biosynthesis by the latter. These results suggest that certain *S. thermophilus* strains are highly recommended to co-culture with high GABA producer for manufacturing GABA-rich fermented milk.

γ-Aminobutyric acid (GABA), a non-protein amino acid, is widely found in plants, microorganisms and vertebrates^1^,^2^. GABA-rich foods that are naturally produced have been popular for decades, and have shown anti-hypertensive effect as an important function^2^,^3^,^4^,^5^,^6^,^7^. In general, GABA content in plant and animal products is very low for delivering any functional benefit in human. Thus, there has been an increasing interest in using high GABA-producing microorganisms for manufacturing GABA-rich fermented milk products such as yogurt and cheese.

Currently, most high GABA producers belong to *Lactobacillus* species, and *Lactobacillus brevis* has been identified as a key species for producing GABA^8^. It has been well documented that glutamic acid decarboxylase (GAD) operon comprise a transcriptional regulator (*gadR*), glutamate decarboxylases (*gadA* or/and *gadB*) and a glutamate/GABA antiporter (*gadC*) in GABA-producing microorganisms^9^. Moreover, high GABA-producing *L. brevis* of plant origin has been isolated from Korean kimchi or other fermented vegetables^8^. Genomic analysis indicated the absence of genes encoding extracellular or cell wall-anchored proteinases in the sequenced *L. brevis* ATCC 367 (a starter culture for beer, sourdough and silage) and *L. brevis* KB290 (an isolate from traditional Japanese fermented vegetable). This may suggest that *L. brevis* of plant origin may not able to survive in milk environments because of its poor proteolytic nature. It is known to us that mammalian milks contain lactose and casein as the major sugar and protein sources, but these are not ideal sources of nutrients for the growth of non-proteolytic lactic acid bacteria (LAB).

GABA-producing LAB shows great promise for manufacturing GABA-rich fermented milk. For instance, milk fermented by *L. casei* Shirota and *Lactococcus lactis* YIT 2027 contained 10 to 12 mg of GABA per 100 mL of fermented milk, this functional food has shown the functionality of lowering the blood pressure in mildly hypertensive patients^5^; *L. helveticus* ND01 yielded 165.11 mg of GABA per 1 kg of fermented milk after 20 h fermentation at 37 °C^10^; *Lactococcus lactis* DIBCA1 and *L. plantarum* PU11 supplemented with 20 mmol/L of glutamic acid produced 144.5 mg of GABA per 1 kg of fermented milk after 48 h fermentation at 37 °C^11^. It is known to us that *Streptococcus thermophilus* and *Lactobacillus delbrueckii* subsp. *bulgaricus* (hereafter *L. bulgaricus*) are important starter microorganisms required for the manufacture of fermented dairy foods such as yogurt and certain cheese varieties^12^,^13^,^14^. In addition, monosodium glutamate (MSG) is normally added to milk as the substrate for manufacturing GABA-rich fermented milk because of low content of free glutamate in milk^8^.

High GABA producer of plant origin may not be able to survive in milk, or may not even ferment milk. Although their viability in milk could be enhanced by adding particular nutrients to milk base, this practice may not be of interest for dairy industry. Although some probiotics or novel LAB strains were adopted as adjunct starters for milk fermentation, conventional dairy starters including *S. thermophilus* and *L. bulgaricus* are required to add into the milk because of the regulations in most countries. Till now, there is very little information on the synergistic effect of high GABA producers and dairy starters. In the present study, we report a new strategy of manufacturing GABA-rich fermented milk by co-culturing high GABA producer with dairy starter including *S. thermophilus* and *L. bulgaricus* in skimmed milk supplemented with MSG, and provide new insights into the effects of dairy starters on the cell viability of *L. brevis* NPS-QW-145 (a high GABA producer; hereafter *L. brevis* 145) and its GABA biosynthesis ability in milk.

## Results

### Two GAD genes were detected in the genome of *L. brevis* 145

**Figure 1:**
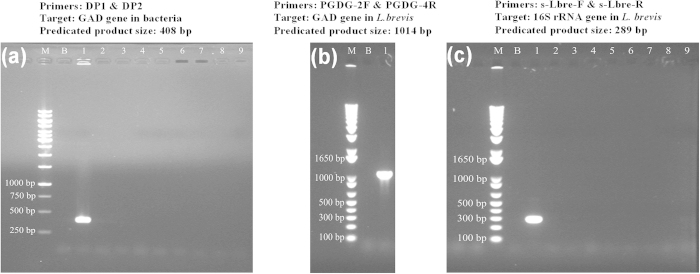
Amplification of GAD gene(s) and 16S rRNA gene from *L. brevis* 145 and eight dairy starters. (**a**) detection of GAD gene using degenerate primers DP1 and DP2; (**b**) amplification of GAD gene(s) in *L. brevis* 145 using degenerate primers PGDG-2F and PGDG-4R; (**c**) specificity of primers s-Lbre-F and s-Lbre-R for amplifying 16S rRNA gene from *L. brevis*. Denotation: M, DNA ladders (Promega 1 kb DNA ladder in Fig. 1a; Invitrogen 1 Kb Plus DNA Ladder in Fig. 1b and Fig. 1c); B, amplification without DNA; Lane 1, *L. brevis* 145; Lane 2, *S. thermophilus* ASCC 1275; Lane 3, *S. thermophilus* ASCC 1303; Lane 4, *S. thermophilus* YI-B1; Lane 5, *S. thermophilus* YI-N1; Lane 6, *S. thermophilus* YI-M1; Lane 7, *L. bulgaricus* ASCC 756; Lane 8, *L. bulgaricus* ASCC 859; Lane 9, *L. bulgaricus* YI-B2.

The amplification result of partial GAD gene (~408 bp) in the eight dairy starters and *L. brevis* 145 is shown in Fig. 1a. Normally, the full length of GAD gene is ~1400 bp. As shown in the Fig. 1a, this gene in all the selected dairy starters including *S. thermophilus* and *L. bulgaricus* was not detected, while it existed in *L. brevis* 145. Moreover, there was no GABA production from these dairy starters when cultured in milk and M17/MRS broth (data not shown). Thus, it was concluded that the GABA was only produced by *L. brevis* 145.

**Figure 2:**
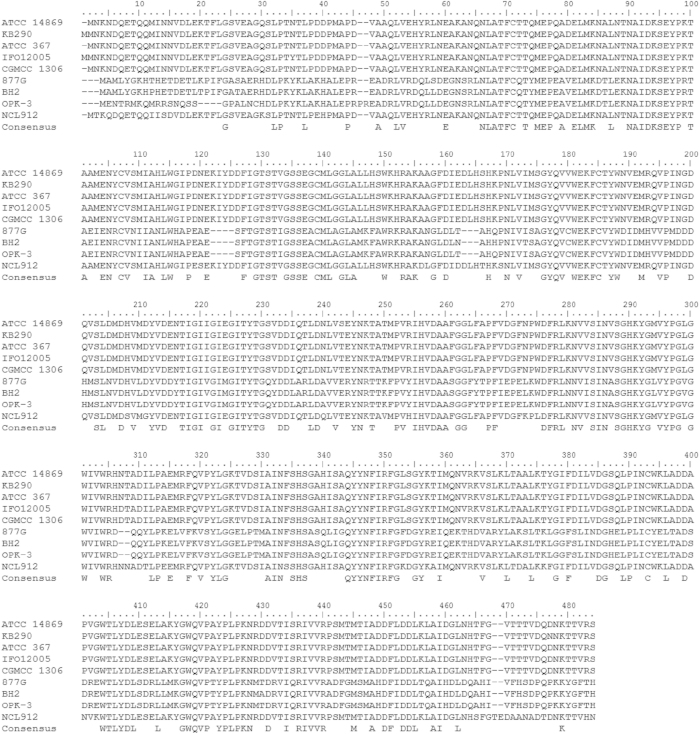
Alignment of the amino acids of full-length glutamate decarboxylases from nine *Lactobacillus brevis* strains. The conserved regions [NAIDKSEYPR(K)TA] and [GWQVPA(T)YPLPKN] were used to design degenerate primers. This figure was generated from BioEdit (version 7.2.5) after ClustalW multiple alignment.

The partial GAD gene from *L. brevis* 145 was successfully amplified and sequenced (Fig. 1b). The size of PCR product was about 1014 bp based on the alignment of amino acids sequence of GAD gene in *L. brevis* (Fig. 2). Interestingly, two GAD genes, *gadA* and *gadB*, were found in *L. brevis* 145 after analyzing the sequences of the PCR product. Excluding the length of degenerate primers PGDG-2R (35 bp) and PGDG-4R (32 bp), the length of the amplified *gadA* and *gadB* was 948 bp and 921 bp, respectively. The GenBank accession numbers for the partial *gadA* and *gadB* sequences of *L. brevis* 145 are KM875632.1 and KM875633.1, respectively. The nucleotides sequences of partial *gadA* and *gadB* showed a similarity of 99% with GAD gene in other *L. brevis* strains (KB290, 877G, CGMCC 1306, ATCC 367, BH2 and IFO 12005). The predicated amino acids sequence of amplified *gadA* (316 aa) only have 164 aa in common with that of amplified *gadB* (307 aa) after ClustalW alignment. Besides the above genetic analysis, we have confirmed high GABA production from this organism^15^. These sequences were further used to design qPCR primers for quantifying the expression of GAD genes in *L. brevis* 145 as shown in Table 1.

### The pH of fermented milks using mixed-cultures or mono-culture

**Figure 3:**
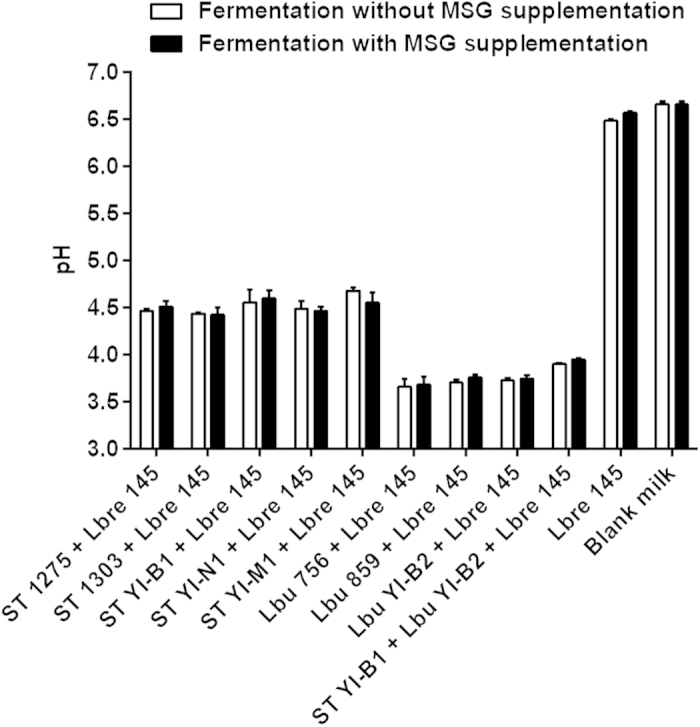
The pH of fermented milks after co-culturing of *L. brevis 145* with *S. thermophilus* or/and *L. bulgaricus*. ST, *S. thermophilus*; Lbu, *L. bulgaricus*; Lbre 145, *L. brevis* 145; Blank milk, 10% (w/v) skimmed milk.

The pH of the fermented milks is shown in Fig. 3. As shown in the Figure, the pH in the milk fermented by *L. brevis* 145 alone was similar to that of the blank milk suggesting that *L. brevis* 145 was not able to ferment milk. Co-culturing of *L. brevis* 145 with *S. thermophilus* in milk after 24 h fermentation at 37 °C resulted in an average pH of ~4.50, whereas co-culturing of *L. brevis* 145 with *L. bulgaricus* showed an average pH of ~3.70. The pH of the milk fermented by three cultures of *L. brevis* 145, *S. thermophilus* YI-B1 and *L. bulgaricus* YI-B2 was ~3.90. It was observed that the pH of the milk fermented by co-cultures of *L. brevis* 145 and *S. thermophilus* or *L. bulgaricus* was not significantly (*P* ≥ 0.05) changed after the supplementation with MSG. This suggests that the addition of MSG did not influence the pH of the milk. Additionally, the pH (~4.50) of milk fermented by *S. thermophilus* and *L. brevis* 145 was similar to that of commercial yogurts. This implies that using *S. thermophilus* and *L. brevis* 145 could be used to produce a yogurt-like product.

### Cell viabilities of *S. thermophilus* and *L. bulgaricus* in milk

**Figure 4:**
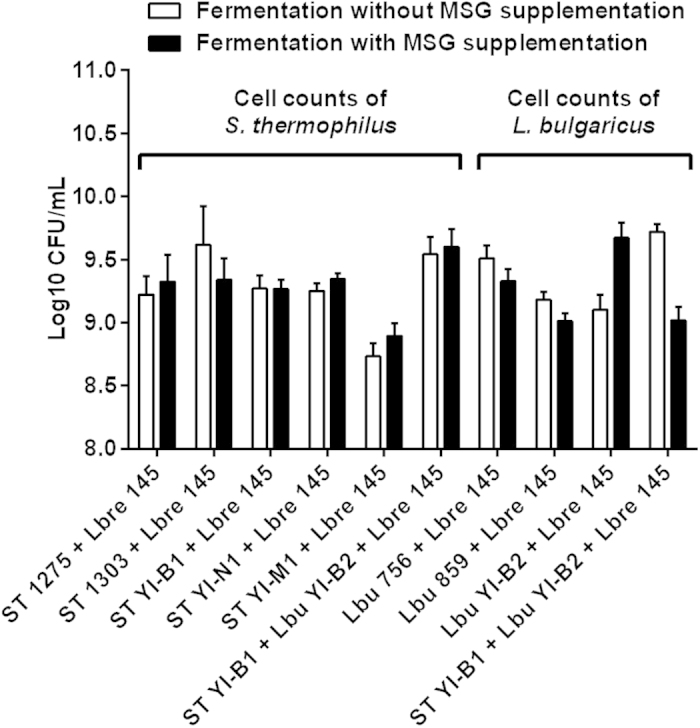
Cell viabilities of *S. thermophilus* and *L. bulgaricus* in fermented milks. ST, *S. thermophilus*; Lbu, *L. bulgaricus*; Lbre 145, *L. brevis* 145.

Cell viabilities of eight dairy starters in fermented milks are shown in Fig. 4. As indicated in the figure, the viabilities of *S. thermophilus* and *L. bulgaricus* co-cultured with *L. brevis 145* in fermented milks were not significantly (*P* ≥ 0.05) changed after the supplementation with MSG. This suggests that MSG supplemented (2 g/L) to milk did not have much influence on the viabilities of both *S. thermophilus* and *L. bulgaricus* cells. Also, the cell counts of both dairy starters were above 8.5 Log_10_ CFU/mL in milk.

### Cell viability of *L. brevis* 145 after co-culturing with dairy *S. thermophilus* or/and *L. bulgaricus* in milk

The primers (s-Lbre-F and s-Lbre-R; Table 1) showed strong specificity for amplifying partial 16S rRNA gene in *L. brevis* 145 (Fig. 1c). The efficiency of this qPCR assay was 98.435%. This indicated that this pair of primers was suitable for qPCR quantitation of *L. brevis* 145 cells and for further gene expression experiments. The equation of standard curve is y = –4.1026x + 52.009 (R^2^ = 0.9851; y, C_t_ value; x, cell counts]. The standard curves showed a good correlation coefficient value (R^2^ = 0.9851), suggesting that the C_t_ values were linear over the range of cell count tested (3.2 × 10^4^ ~ 3.2 × 10^9^ CFU/mL). The analysis of the melting curves did not show the formation of non-specific fragments or primer-dimers indicating that the qPCR assay was accurate and reproducible.

**Figure 5:**
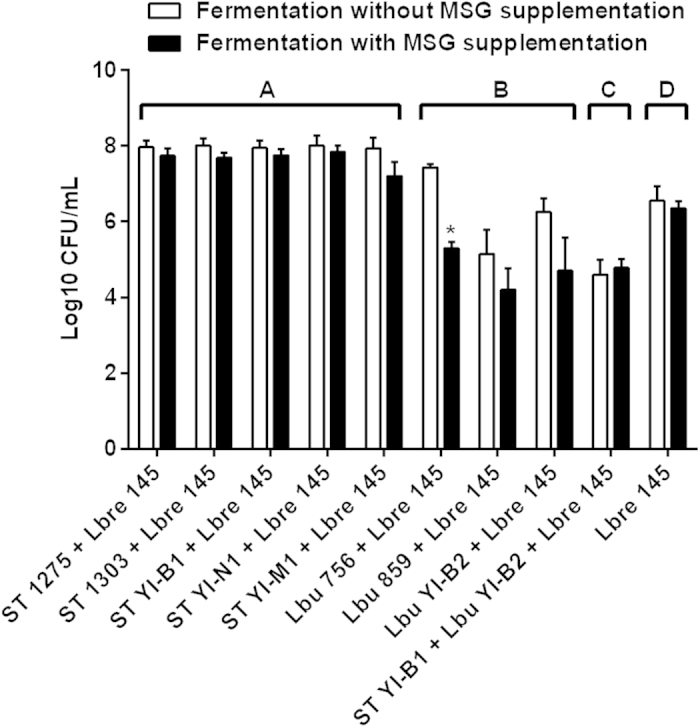
Cell counts of *L. brevis* 145 in fermented milks. ST, *S. thermophilus*; Lbu, *L. bulgaricus*; Lbre 145, *L. brevis* 145. Star (*P < 0.05) is for the comparison of data between fermentation with and without the supplementation of MSG; Capital letters (A, B, C and D) are designated to indicate the significance of the group data of *L. brevis* counts, the same letter among each group indicates no significance (*P* ≥ 0.05). The initial cell counts of *L. brevis* 145 after inoculation in milk was ~3 × 10^7^ CFU/mL (7.48 Log_10_ CFU/mL).

The viability of *L. brevis* 145 in milk during co-culturing is shown in Fig. 5. Before the fermentation, the initial counts of *L. brevis* 145 cells in milk were ~3 × 10^7^ CFU/mL (~7.48 Log_10_ CFU/mL). However, the counts of this strain decreased to ~6.50 Log_10_ CFU/mL after 24 h of fermentation (Fig. 5). This indicates that viability of *L. brevis* 145 was not maintained in milk during fermentation. In general, it was observed that the viability of *L. brevis* 145 decreased slightly but not significantly (*P* ≥ 0.05) after supplementation with MSG to milk, except the fermentation using co-cultures of *L. brevis* 145 and *L. bulgaricus* ASCC 756. Interestingly, the average cell counts of *L. brevis* 145 after co-culturing with *S. thermophilus* was ~7.90 Log_10_ CFU/mL, which was significantly (*P* < 0.01) higher than that of co-culturing with *L. bulgaricus*, co-culturing with both *S. thermophilus* and *L. bulgaricus*, and the control fermentation with only *L. brevis* 145. For co-culturing with *L. bulgaricus*, the viability of *L. brevis* 145 decreased significantly (*P* < 0.01) as compared with the control using only *L. brevis* 145. Thus, it was suggested that the presence of *L. bulgaricus* in co-culture with *L. brevis* 145 had a negative effect on the viability of *L. brevis* 145.

### GABA yield and residual MSG content in fermented milks

The content of GABA and residual MSG in milk supplemented with or without MSG is shown in Table 2. As shown in the table, GABA was not detected in the milk fermented by only *L. brevis* 145. This is mainly because the milk composition was not able to support the growth of this organism (Fig. 3), which was also evidenced by the decreased viability of this strain in milk grown alone (Fig. 5). However, after co-culturing with certain *S. thermophilus* strains (ASCC 1275, YI-B1 and YI-N1), GABA production was increased in milk supplemented with MSG after 24 h fermentation. Co-culturing of *S. thermophilus* YI-B1 and *L. brevis* 145 in milk containing 2 g/L of MSG yielded the highest level (~314 mg per 1 kg of fermented milk) of GABA after 24 h of fermentation. Till now, this may be the highest known amount of GABA content in fluid milk products that were fermented by LAB^5^,^10^,^11^.

MSG content at a high level in milk may not be appreciated because of its flavor. In the present study, it was found that GABA production did not correlate with the reduction in MSG level (Table 2). Hence, the level of MSG supplemented to milk could be modified. In general, the level of residual MSG in milk fermented by *S. thermophilus* and *L. brevis* 145 was lower than that by *L. bulgaricus* and *L. brevis* 145. Because there was very limited GABA production converted from MSG, the high glutamate in milk fermented by *L. bulgaricus* and *L. brevis* 145 may be due to the better extracellular proteolytic activity of *L. bulgaricus* than that of *S. thermophilus*. This also suggests that *L. bulgaricus* may have obtained sufficient glutamate from milk proteins after hydrolysis. In addition, MSG content in milk fermented by *S. thermophilus* YI-N1 and *L. brevis* 145 was the lowest, which indicates that *S. thermophilus* YI-N1 may have utilized more MSG than that by other selected dairy starters. Thus, *S. thermophilus* strains could be used for reducing the MSG level in fermented milk.

### GAD gene expression in *L. brevis* 145

We wanted to find a suitable housekeeping gene in *L. brevis* for normalization; however, the expressed stable genes in *L. brevis* including *tuf* (elongation factor Tu)^9^, *proC* (amino acid biosynthesis) and *rpoB* (RNA polymerase)^16^ are not specific for *L. brevis*. These genes also exist in *S. thermophilus*. Clear bands were observed in agarose gel after electrophoresis when amplification was carried out for five strains of *S. thermophilus* using the primers reported in above studies^9^,^16^. Thus, 16S rRNA gene was used as a housekeeping gene for real-time qPCR assay using the primers exhibited in Table 1 and its efficiency was assessed as well. The efficiencies of this qPCR assay using 16S rRNA gene, *gadA* and *gadB* were 91.78%, 99.54% and 101.39%, respecitively.

**Figure 6:**
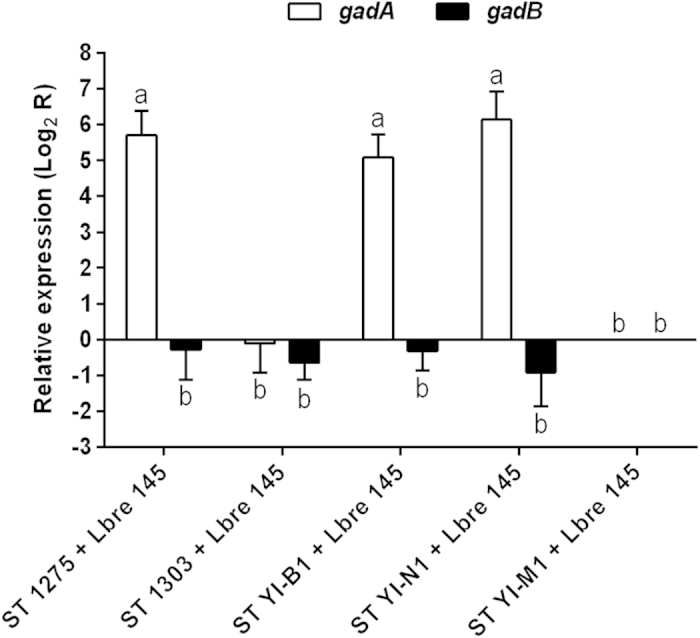
Relative gene expression of *gadA* and *gadB* in *L. brevis* 145 after co-cultured with *S. thermophilus* in milk supplemented with 2 g/L of MSG. The levels of *gadA* and *gadB* mRNA from *L. brevis* 145 after co-cultured with *S. thermophilus* YI-M1 was used as a reference for comparision. Comparative critical threshold method (R = 2^–ΔΔCт^) was carried out for data analysis of three indendent exeriments, a positive value indicates up-regulation while a negative value indicates down-regulation. Lowercase letters (a & b) are designated to indicate the significance of *gadB* and *gadA* mRNA levels, the same letter above/below each bar indicates no significance (*P* ≥ 0.01). ST, *S. thermophilus*; Lbre 145, *L. brevis* 145.

The result of qPCR quantitation of *gadA* and *gadB* mRNA level in *L. brevis* 145 is shown in Fig. 6. Interestingly, it was observed that only the *gadA* mRNA level in *L. brevis* 145 was significantly (*P* < 0.01) up-regulated by certain *S. thermophilus* strains (ASCC 1275, YI-B1 and YI-N1) as compared with other two *S. thermophilus* strains (ASCC 1303 and YI-M1), whereas the *gadB* mRNA level in *L. brevis* 145 was not regulated by all selected *S. thermophilus* strains. The improved *gadA* mRNA level may suggest an enhanced GABA biosynthesis in *L. brevis* 145 resulting in an increased GABA production after co-culturing with *S. thermophilus* ASCC 1275, YI-B1 and YI-N1 in milk supplemented with 2 g/L of MSG (Table 2).

## Discussion

*S. thermophilus* and *L. bulgaricus* are two common dairy starters that are highly recommended for the manufacture of yogurt and several type of cheeses. Thus, in this study the above starters were co-cultured with *L. brevis* 145 for making GABA-rich fermented milk. Also, MSG was supplemented as the substrate for GABA production. However, MSG is additional sodium salt in milk and its effects on milk fermentation needs to be demonstrated. It was observed that MSG supplemented at the level of 2 g/L did not show much effect on the pH of milk (Fig. 3), cell viabilities of *S. thermophilus* and *L. bulgaricus* (Fig. 4), and the viability of *L. brevis* 145 (Fig. 5). These results suggest that supplementation with MSG at 2 g/L or below this level to milk base may be an option for making functional fermented milk.

Without MSG supplementation, GABA production from *L. brevis* 145 was very low when co-cultured with *L. bulgaricus*, whereas GABA was not detected when co-cultured with *S. thermophilus* (Table 2). However, MSG supplemented at 2 g/L in milk improved the GABA production greatly from *L. brevis* 145 when only co-cultured with certain *S. thermophilus* strains, while its production was not significantly increased during co-culturing with *L. bulgaricus* (Table 2). Thus, it appears that MSG supplementation was necessary for an improved GABA production from *L. brevis* 145. However, further documentation on the flavor of this fermented milk is necessary because of the introduction of MSG in milk. Interestingly, it was found that *S. thermophilus* could utilize more MSG than that of *L. bulgaricus*. This may be of particular interest for dairy industry.

Use of *L. bulgaricus* for co-culturing with *L. brevis* 145 in milk may not be ideal because of the generation of low counts of *L. brevis* 145 by this species (Fig. 5). This is possibly due to competition and because they belong to the same *Lactobacillus* genus^17^. However, use of *S. thermophilus* could be a promising option due to its ability to maintain the viability of *L. brevis* 145 in milk. A previous study revealed that formic acid, folic acid and fatty acids released from *S. thermophilus* supported the growth of *Lactobacillus* genus in milk^18^. Moreover, dairy *S. thermophilus* possesses good extracellular proteolytic property and could also supply *L. brevis* 145 with sufficient amino acids or peptides^19^,^20^. This may explain that *S. thermophilus* was able to support the growth of *L. brevis* 145 in milk during co-culturing. However, only certain strains of *S. thermophilus* (ASCC 1275, YI-B1 and YI-N1) improved the GABA yield from *L. brevis* 145 (Table 2), which was closely associated with an increased *gadA* mRNA level in *L. brevis* 145 when co-cultured with above strains (Fig. 6). This implies that the GABA biosynthesis in *L. brevis* 145 could be up-regulated by certain *S. thermophilus* strains.

Interestingly, *gadA* and *gadB* were found to be independently conserved in *L. brevis* based on their amino acids sequences, and some strains only possessed *gadA* (NCL912 and IFO12005) or *gadB* (BH2, 877G and OPK-3), while some strains (KB290, ATCC367, BSO 464, AG48, EW and DmCS_003) may have both genes in their genomes. It has been demonstrated that *gadA* in previously studied *L. brevis* strains (NCL912 and IFO12005) and *gadB* in *L. brevis* strains (BH2, 877G and OPK-3) have shown their capability of producing high amount of GABA in their host. This indicates that both two glutamate decarboxylases are functional, and may exhibit similar enzymatic activity because they may possess the same core conformation. Moreover, certain *S. thermophilus* strains (ASCC 1275, YI-B1 and YI-N1) regulated the *gadA* expression in *L. brevis* 145, while the level of *gadB* mRNA transcripts was not affected by the former. Other *S. thermophilus* strains (ASCC 1303 and YI-M1) were not able to influence the level of both *gadA* and *gadB* transcripts. This may be related with the strain-specific interactions between *S. thermophilus* and *L. brevis* 145 regarding to the metabolism of purine, amino acid and long-chain fatty acid^18^. It was found that the location of *gadA* and *gadB* in the genome of sequenced *L. brevis* strains (KB290, ATCC367, BSO 464, AG48, EW and DmCS_003) was not close to each other, but only one GAD gene (*gadA* or *gadB*) was found in the *gad* operon. This suggests that there may be different mechanism for the regulation of *gadA* and *gadB* gene expression in their hosts. This merits further investigation on the regulation by certain *S. thermophilus* strains.

### Concluding remarks

In this study, two glutamate decarboxylase gene, *gadA* and *gadB*, were found in high GABA-producing *L. brevis* 145. However, this organism was not able to ferment milk. It was observed that all the selected dairy *S. thermophilus* strains, but not *L. bulgaricus*, improved the viability of *L. brevis* 145 when co-cultured in milk. Only certain *S. thermophilus* strains improved GABA production from *L. brevis* 145, which was evidenced by the increased *gadA* mRNA transcripts in the latter. Moreover, co-cultures of *S. thermophilus* and *L. brevis* 145 utilized more MSG than co-cultures of *L. bulgaricus* and *L. brevis* 145 suggesting the use of *S. thermophilus* for reducing MSG content if supplemented in milk. This study provides a new insight of using *S. thermophilus* for co-culturing with high GABA producer of plant origin for manufacturing GABA-rich fermented milk.

## Methods

### Bacterial strains and culture conditions

Non-dairy starter *L. brevis* NPS-QW-145, a high GABA-producing strain isolated from Korean kimchi, was used in this study as a model of high GABA producer^15^. Eight dairy starters (Table 1) were used for co-culturing with this organism. *Lactobacillus* strains were activated in Difco^TM^ lactobacilli MRS broth (BD Company, MD, USA), while *S. thermophilus* strains were cultivated in M17 broth (BD Company). Working cultures were propagated three times consecutively using 1% inoculation in the above medium (MRS or M17) at 37 °C for 18 h.

### Alignment of the amino acids of glutamic acid decarboxylase (GAD) from *L. brevis*

In order to amplify the GAD gene from *L. brevis* 145, degenerated primers were designed according to the conserved regions of this enzyme from the species of *L. brevis*. The full-length sequences of amino acids of GAD from *L. brevis* strains were downloaded from the database of the National Center for Biotechnology Information (NCBI), and were aligned using BioEdit software (version 7.2.5). The conserved region [NAIDKSEYPR(K)TA] was used for designing the forward primer, whereas another conserved sequence [GWQVPA(T)YPLPKN] was for designing the reverse primer (Fig. 2). The degenerate primers, PGDG-2F and PGDG-4R, are shown in Table 1.

### Amplification of GAD gene in selected dairy starters and *L. brevis* 145

After growing the selected bacteria in the respective medium (MRS or M17), genomic DNAs from eight dairy starters and *L. brevis* 145 were isolated and purified by using ChargeSwitch^®^ gDNA Mini Bacteria Kit (Invitrogen, Carlsbad, CA, USA) according to the manufacturer’s instructions. One pair of degenerate primers DP1 and DP2, PGDG-2F and PGDG-4R (Table 1) was applied for amplification of partial GAD gene using AmpliTaq^®^ Gold 360 master mix (Applied Biosystems, Foster, CA, USA). Based on the manufacturer’s instruction, the PCR reaction volume (25 μL) included 12.5 μL of master mix, 0.5 μL of each primer (10 μM), 2 μL (~1 ng) of DNA template and 8.5 μL of DNase-free water. The amplification was carried out in a GeneAmp^®^ PCR system 2700 (Applied Biosystems) with 35 cycles (94 °C for 30 s, 60 °C for 30 s, and 72 °C for 90 s) for partial GAD gene. Agarose gel (1%; w/v) electrophoresis was carried out for all PCR products. The size of the PCR products was ~1014 bp.

### Sequencing of GAD gene in *L. brevis* 145

After amplification of partial GAD gene from *L. brevis* 145, the PCR products from agarose gel were excised and purified according to the manufacture’s instruction of S.N.A.P.^TM^ Gel Purification Kit (Invitrogen). The purified DNAs were ligated with pCR™4-TOPO® TA vector based on the manufacturer’s instructions of the TOPO^®^ TA Cloning^®^ Kit (Invitrogen), and the ligated plasmids were further transformed into One Shot^®^ TOP10 Chemically Competent *Escherichia coli* (Invitrogen). After white/blue agar screening and colony-PCR amplification, the plasmids from positive colony were extracted and the amplification of inserted sequence was carried out using M13 primers. Then, PCR products were purified and sequenced in 3130xl Genetic Analyzer (Applied Biosystems) using BigDye® Terminator v3.1 Cycle Sequencing Kit (Applied Biosystems). Sequence reads were further assembled and aligned. The sequence of partial GAD gene was used for designing qPCR primers for quantifying the expression of GAD gene in *L. brevis* 145.

### Mixed cultures and culture conditions for skimmed milk fermentation

The cell counts of the working cultures were enumerated on MRS or M17 agar plates using plate counting method prior to inoculation in skimmed milk. The initial cell count for *L. brevis* 145 was ~1 × 10^9^ CFU/mL, while the cell counts for *S. thermophilus* and *L. bulgaricus* were ~1 × 10^9^ CFU/mL and ~1 × 10^8^ CFU/mL, respectively.

Milk fermentation using cultures with *L. brevis* alone, or in co-culture with single dairy starter or in co-culture with both *S. thermophilus* YI-B1 and *L. bulgaricus* YI-B2 were performed. Monoculture fermentation of *L. brevis* 145 was carried out in 10% (w/v) skimmed milk supplemented with or without 2 g/L of MSG at 3% (v/v) inoculation level. For co-culturing of *L. brevis* 145 with one dairy starter in skimmed milk with or without MSG, the inoculation level of *L. brevis* 145 was 3% (v/v) and that of the dairy starter was 1% (v/v). For co-culturing of *L. brevis* 145 with two different dairy starters, *S. thermophilus* YI-B1 and *L. bulgaricus* YI-B2 were used as conventional starters, while *L. brevis* 145 was used as an adjunct culture. The inoculation level of each of the *S. thermophilus* YI-B1 and *L. bulgaricus* YI-B2 was 0.5% (v/v), while the inoculation level of *L. brevis* 145 was 3% (v/v). All the fermentation experiments were carried out at three occasions under static condition at 37 °C for 24 h.

### Measurement of the pH of fermented milks

The pH of the fermented milk was measured using Orion Model 250A portable pH Meter (Thermo Scientific, Wilmington, DE, USA).

### Selective enumeration of *S. thermophilus* and *L. bulgaricus* in milk

After milk fermentation, enumeration of *S. thermophilus* and *L. bulgaricus* was carried out using selective medium as previously described^21^. Briefly, the viable counts of above two species were enumerated by plating aliquots of serial dilutions on M17 agar and MRS agar (pH 5.2) plates, respectively. The M17 agar plates for *S. thermophilus* were incubated aerobically at 37 °C for 24 h, while MRS agar (pH 5.2) plates for enumerating *L. bulgaricus* were anaerobically kept at 45 °C for 48 h, followed by counting colonies.

### Real-time qPCR assay for measuring the cell counts of *L. brevis* 145 in milk

Since there was no available selective medium for enumeration of *L. brevis* cells, real-time qPCR was used for assessing the cell count of *L. brevis* 145 after co-culturing with dairy starters in skimmed milk. Genomic DNA was extracted using the bead-beating extraction method as previously described^22^. Briefly, 200 μL of fermented milk, 0.40 g of glass beads (0.1 mm diameter; BioSpec Products, Bartlesville, OK, USA) and 600 μL of extraction solution [500 mM of NaCl, 50 mM of Tris, 50 mM of EDTA, 4% SDS (w/v), pH 8.0], and 200 μL of phenol/chloroform/isoamyl alcohol (25:24:1) were added into 2-mL microcentrifuge tubes, followed by disrupting the cells in a BR-2000 Vortexer (Bio-Rad, Hercules, CA, USA) at the highest speed for 5 min. Then, the mixture was separated by centrifugation (12,000* *×* g*; 15 min; 4 °C), and upper aqueous phase containing DNAs was transferred to a new tube. The aqueous phase was washed twice with 600 μL of phenol/chloroform/isoamyl alcohol (25:24:1), and the DNAs were precipitated by adding sodium acetate and isopropanol followed by centrifugation (12,000* *×* g*; 15 min; 4 °C). The DNA pellet was washed with pre-cooled 75% ethanol. Finally, the precipitated DNAs were dissolved in 30 μL of TE buffer and stored at −30 °C for further analysis.

The amplification was carried out in a StepOnePlus^TM^ Real-Time PCR system (Applied Biosystem). For amplification, 25 μL reaction mixture contained 12.5 μL of SYBR Green master mix, 1 μL of 10 mM of each primer – s-Lbre-F and s-Lbre-R (Table 1), and 2 μL of template DNA. Real-time qPCR was performed with initial denaturation at 95 °C for 5 min, followed by 40 cycles of denaturation at 95 °C for 10 s, primer annealing 55 °C for 30 s, and extension at 72 °C for 30 s. At the end of PCR run, melting curve analysis was carried out from 60 °C to 95 °C (0.5 °C/s) for detection of primer-dimers. The efficiency of this qPCR assay using primers s-Lbre-F and s-Lbre-R was examined by 10-fold serially diluting the genomic DNA from *L. brevis* 145 cultures and 5 dilutions were used for qPCR assay. The standard curve generated from threshold cycle (C_t_) value and viable cell counts of *L. brevis* ranging from 3.2 × 10^4^ CFU/mL to 3.2 × 10^9^ CFU/mL was prepared in milk as well. The bead-beating extraction procedure was also carried out for isolating the DNA from *L. brevis* 145 diluted in skimmed milk. Real-time qPCR amplification was carried out in duplicates for each sample and three independent experiments were carried out.

### Reversed-phase HPLC analysis of glutamate and GABA

Carrez solutions were used to remove milk proteins before reversed phase HPLC analysis for glutamate and GABA^23^. Briefly, one gram of fermented milk was added into 4.0 mL of distilled water, followed by addition of 0.25 mL of Carrez I solution (0.25 M potassium ferocyanide) and 0.25 mL of Carrez solution II (0.50 M zinc acetate). Then, the mixture was thoroughly mixed, and kept for 30 min at room temperature until the complex formation and precipitation of milk proteins, followed by centrifugation at 25 °C and 5,000 × *g* for 30 min. Supernatants were collected and filtered through 0.20 μm millipore filter. Filtrates were then freeze-dried, followed by re-dissolving in double distilled water and removing residues by centrifugation at 4 °C and 5,000 × *g* for 30 min. The clear supernatants with free amino acids were again filtered through 0.20 μm millipore filter. Dansyl derivatization of free amino acids including MSG and GABA was carried out, followed by HPLC analysis of dansyl amino acids as previously described^15^.

### RNA isolation and cDNA synthesis

A modified hot SDS/hot phenol extraction method was used to obtain high quality RNA from Gram-positive bacteria^24^. Approximately 4 mL of fermented milk was re-suspended in 36 mL of ice-cold sterile water containing 1% (v/v) β-mercaptoethanol, followed by addition of 4 mL of ice-cold ethanol and vortexing for 1 min. Milk proteins were removed by centrifugation at 4 °C and 233 × g for 5 min, and supernatants were collected and centrifuged at 4 °C and 5,000 × g for another 10 min. The harvested bacterial pellet was resuspended thoroughly in 1 mL of RNAlater^TM^ buffer (Qiagen, Limburg, The Netherlands) and incubated at room temperature for 5 min. Then, the bacterial suspension was centrifuged again and the cell pellet was washed with ice-cold sterile water containing 1% (v/v) β-mercaptoethanol to remove residual salts. Cell pellets were resuspended in 600 μL of lysis buffer consisting of 1% (v/v) β-mercaptoethanol and 0.5 mg/mL lysozyme (Sigma-Aldrich, St. Louis, MO) in TE buffer, and 200 mg of glass beads (0.1 mm diameter; BioSpec Products) was added into the suspension. Then, the suspension was vortexed in a BR-2000 Vortexer (Bio-Rad) at the highest speed for 5 min. After that, 60 μL of 10% (w/v) SDS solution and 66 μL of 1 M sodium acetate (pH 5.2) was mixed with the lysate. Additionally, 600 μL of phenol:chloroform:isoamyl alcohol (25:24:1) was added, mixed and incubated at 64 °C for 10 min. The tubes were inverted several times every 2 min. The mixture was chilled in an ice bath for 5 min and centrifuged at 4 °C and 21,000 × g for 10 min. The aqueous layer was transferred and washed with equal volume of phenol:chloroform:isoamyl alcohol (25:24:1) twice and centrifuged at 4 °C and 21,000 × g for 5 min. The aqueous layer was transferred to 1.5 mL Eppendorf tubes, and RNA was precipitated with ethanol by adding 1/10 volume of 3 M sodium acetate (pH 5.2) and 2 volume of cold ethanol to each tube. The samples were mixed and incubated at −30 °C overnight. The RNA was pelleted by centrifuging at 4 °C and 21,000 × g for 25 min followed by washing with ice-cold 75% ethanol. The pellet was re-suspended in 20 μL of RNase-free water. RNAs isolated after DNase I (Invitrogen) treatment were further converted into cDNAs by reverse transcription according to the manufacture’s instruction of High-Capacity RNA-to-cDNA^TM^ Kit (Applied Biosystems).

### Real-time qPCR quantitation of *gadA* and *gadB* mRNA transcripts in *L. brevis* 145

The amplification was also carried out in a StepOnePlus^TM^ Real-Time PCR system (Applied Biosystem). The 25 μL reaction mixture contained 12.5 μL of SYBR Green master mix, 1 μL of 10 mM of each primer for 16S rRNA gene (reference gene) and GAD genes (Table 1), and 2 μL of template cDNA. Real-time qPCR was carried out with initial denaturation at 95 °C for 10 min, followed by 40 cycles of denaturation at 95 °C for 15 s, and annealing and extension at 60 °C for 60 s. The efficiency of this qPCR assay using three pair of primers including s-Lbre-F and s-Lbre-R, gadA-757F and −945R, gadB-364F and −499R (Table 1) was also examined by 10-fold serially diluting the genomic DNA from *L. brevis* 145 cultures and 5 dilutions were used for the qPCR assay. RT-qPCR analysis was carried out for each sample in duplicate and all the experiments were replicated three times.

### Statistical analysis

All presented data in the bar charts and tables correspond to means ± standard deviation. Significant difference (*P* < 0.05 or *P* < 0.01) among the groups was carried out by one-way analysis of variance (ANOVA) using IBM SPSS Statistics 20.0 version.

## Additional Information

**How to cite this article**: Wu, Q. *et al.* Dairy *Streptococcus thermophilus* improves cell viability of *Lactobacillus brevis* NPS-QW-145 and its γ-aminobutyric acid biosynthesis ability in milk. *Sci. Rep.* **5**, 12885; doi: 10.1038/srep12885 (2015).

## Figures and Tables

**Table 1:** Bacterial strains and primers used in this study.

Starter bacteria for milk fermentation		
Species	Strain ID	Origin
*Streptococcus thermophilus*	ASCC 1275	Australian Starter Culture Research Center
	ASCC 1303	Australian Starter Culture Research Center
	YI-B1	Commercial yogurt isolate
	YI-N1	Commercial yogurt isolate
	YI-M1	Commercial yogurt isolate
*Lactobacillus delbrueckii* subsp. *bulgaricus*	ASCC 756	Australian Starter Culture Research Center
	ASCC 859	Australian Starter Culture Research Center
	YI-B2	Commercial yogurt isolate
*Lactobacillus brevis*	NPS-QW-145	High GABA producer isolated from Korean kimchi
**Primers for PCR amplification**		
**Name**	**Sequences (5′ to 3′)**	**Reference**
PGDG-2F	AAYGCSATYGATAAATCSGARTAYCCTMRGACCGC	This study
PGDG-4R	TTYTTTGGYARKGGATAKGYSGGRACYTGCCA	
DP1	ggtacatctacaattggttcttctgaRgcNtgYatg	25
DP2	aaaccaccagaagcagcRtcNacRtgNat	
s-Lbre-F	ATTTTGTTTGAAAGGTGGCTTCGG	26
s-Lbre-R	ACCCTTGAACAGTTACTCTCAAAGG	
gadA-757F	CAGGTTACAAGACGATCATGC	This study
gadA-945R	ATACTTAGCCAGCTCGGACTC	
gadB-364F	GGACAATACGACGACTTAGC	This study
gadB-499R	CTTGAGCTCGGGTTCAATAA	

**Table 2:** GABA production and residual MSG in fermented milks after fermentation at 37 °C for 24 h.

Strains/Control	MSG supplementation (g/L)	Residual MSG (mg per 1 kg of fermented milk)	GABA production (mg per 1 kg of fermented milk)
Blank milk	0	43.90 ± 4.48	N.D.
	2	2094 ± 49.71	N.D.
Lbre 145	0	53.62 ± 3.86	N.D.
	2	2023 ± 100.52	N.D.
ST 1275 + Lbre 145	0	17.02 ± 1.26	N.D.
	2	1208.46 ± 94.19	265.57 ± 34.13
ST 1303 + Lbre 145	0	8.93 ± 2.76	N.D.
	2	1628.13 ± 10.75	25.67 ± 2.00
ST YI-B1 + Lbre 145	0	17.27 ± 1.07	N.D.
	2	1156.70 ± 69.52	314.97 ± 14.45
ST YI-N1 + Lbre 145	0	14.84 ± 1.51	N.D.
	2	985.07 ± 12.33	230.53 ± 34.05
ST YI-M1 + Lbre 145	0	26.92 ± 5.86	N.D.
	2	1426.62 ± 22.97	26.23 ± 0.83
Lbu 756 + Lbre 145	0	330.35 ± 27.62	6.58 ± 0.62
	2	1987.70 ± 48.85	9.79 ± 0.79
Lbu 859 + Lbre 145	0	235.75 ± 3.12	13.53 ± 0.06
	2	1881.44 ± 126.07	11.00 ± 1.26
Lbu YI-B2 + Lbre 145	0	276.23 ± 38.16	10.53 ± 5.22
	2	1827.17 ± 139.44	10.32 ± 2.30
ST YI-B1 + Lbu YI-B2 + Lbre 145	0	130.76 ± 5.68	14.84 ± 1.19
	2	1366.79 ± 53.32	19.52 ± 1.45

N.D., not detectable; ST, *S. thermophilus*; Lbu, *L. bulgaricus*; Lbre, *L. brevis 145*; Blank milk, 10% (w/v) skimmed milk. All the glutamate detected in milk was calculated into residual MSG for comparision.
